# The correlation between the styloid process length, angle, head shape, and eagle syndrome based on high-resolution CT three-dimensional reconstruction: a retrospective study

**DOI:** 10.3389/fmed.2025.1682569

**Published:** 2025-10-02

**Authors:** Jiaqi Duan, Fang Wu, Linfeng Liu, Jianxia Xu, Xiaozhong Zheng, Shufeng Fan, Qinpan Rao

**Affiliations:** ^1^The Second School of Clinical Medicine, Zhejiang Chinese Medical University, Hangzhou, Zhejiang, China; ^2^Department of Radiology, The Second Affiliated Hospital of Zhejiang University of Traditional Chinese Medicine, Hangzhou, Zhejiang, China; ^3^Department of Radiology, The Songyang County Traditional Chinese Medicine Hospital, Lishui, Zhejiang, China

**Keywords:** eagle syndrome, head shape, styloid process, HRCT, logistic regression analysis

## Abstract

**Objective:**

A comprehensive evaluation index (R value) based on the styloid process (SP) length and spatial angle orientation was constructed to explore the imaging and clinical features of Eagle Syndrome, and assess its diagnostic value in the identification of Eagle Syndrome.

**Methods:**

A retrospective analysis was conducted on the high-resolution CT (HRCT) three-dimensional reconstruction maximum intensity projection (MIP), with a data of 57 clinically diagnosed Eagle Syndrome (ES group) and 49 Non Eagle Syndrome (NES group). The bilateral SP length, inward angulation, and forward tilt angle were measured. A comprehensive evaluation index, the R value, was introduced based on the head shape. Independent two-sample t-tests were used to compare the differences in parameters between the two groups, and ROC curve analysis was performed to assess the diagnostic efficacy of the R value. Finally, binary logistic regression was employed to validate the stability of the model.

**Results:**

The ES Group exhibited significantly higher parameters compared to the NES Group in terms of SP length (left: 34.19 ± 5.14 mm, right: 34.13 ± 6.40 mm), inward angulation (left: 24.29° ± 3.09°, right: 22.22° ± 3.18°), and forward tilt angle (left: 28.39 ± 2.76°, right: 28.29 ± 2.72°). The ROC curve analysis of the R value showed that the left side had a AUC of 0.86 (95% CI, 0.79–0.93), with an optimal cutoff value of 2.85, sensitivity of 82.5%, and specificity of 79.6%. The right side had an AUC of 0.82 (95% CI, 0.74–0.90), with an optimal cutoff value of 2.72, sensitivity of 78.9%, and specificity of 75.5%. The binary logistic regression results demonstrated that the R value exhibits excellent discriminative ability in the diagnosis of Eagle Syndrome. In particular, when the left R > 2.85 and/or right R > 2.72, Eagle Syndrome should be strongly suspected, and a precise diagnosis should be made in combination with clinical symptoms.

**Conclusion:**

Eagle Syndrome is closely related to the length and angle of the SP. The R value, as a composite evaluation index integrating key anatomical parameters such as length and angle, demonstrates high diagnostic efficacy and significant clinical utility. Moreover, R value (The left R > 2.85 and/or right R > 2.72) can be applied as quantitative reference criteria for diagnosis.

## 1 Introduction

Eagle Syndrome, also known as stylohyoid syndrome, refers to a condition where abnormal SP length, morphological variations, or calcification of the stylohyoid ligament compresses the surrounding soft tissues, leading to a range of clinical symptoms (1, 2). In 1937, Watt Eagle first systematically described this disease and proposed the view that “any SP longer than 25 mm could be pathogenic.” Although this syndrome is named after Watt Eagle, he was not the first to discover the disease. His significant contribution was linking anatomical variations with a cluster of clinical symptoms and estimating that only about 4% of individuals with an elongated SP ultimately develop symptoms (3). The clinical presentation of Eagle Syndrome is diverse, with typical symptoms including throat pain, a foreign body sensation in the throat, dizziness when turning the neck, anterior-lateral neck pain, and radiating ear pain. Due to the lack of specificity, it is often misdiagnosed as chronic tonsillitis, glossopharyngeal neuralgia, or temporomandibular joint disorders, resulting in a prolonged period without accurate diagnosis or effective treatment (4). Most patients exhibit a gradual onset of unilateral jaw angle pain, which may radiate to the head, face, or neck, and in severe cases, vascular symptoms may occur due to compression of the carotid artery (5, 6). Some patients also show significant deep tenderness in the tonsillar fossa, which can be temporarily relieved by local infiltration of lidocaine, providing certain diagnostic clues.

Imaging studies play a crucial role in the diagnosis of this disease. Although conventional X-ray films are simple, economical, and practical for preliminary assessment of SP length, their two-dimensional imaging nature limits the accurate determination of the spatial orientation of the SP and its relationship with surrounding tissues (7). Additionally, the low resolution of soft tissues makes it easy to miss critical signs such as calcification at the styloid tip or compression of neurovascular structures, especially in cases of underdevelopment, elongation, or segmentation of the SP, which may lead to misinterpretation due to poor visualization or structural overlap. Furthermore, X-ray films have limited visibility of the styloid base, and measurements often rely on subjective experience, making it difficult to ensure accuracy and reproducibility. In contrast, high-resolution CT combined with various three-dimensional post-processing techniques significantly enhances diagnostic accuracy (8, 9). Through multi-planar reconstruction (MPR), it is possible not only to measure the SP length precisely but also to evaluate spatial parameters such as the inward angulation and forward tilt angle. Even in cases of tilted patient positioning, MPR images can adjust the baseline and measurement angles to obtain highly reproducible and accurate spatial data. Therefore, HRCT three-dimensional reconstruction is currently regarded as the “gold standard” for diagnosing Eagle Syndrome (10).

Research has shown that the normal SP length in the general population typically ranges from 20 to 30 mm, with approximately 4 to 28% of individuals having elongated SP, but only 4 to 10% of these individuals exhibit clinical symptoms. Current diagnostic standards mainly rely on imaging combined with clinical symptoms. However, traditional diagnostic methods place too much emphasis on the absolute length of the SP, neglecting the impact of its spatial orientation (such as inward angulation and forward tilt), which may result in missed diagnoses of patients with normal SP length but abnormal orientation. Domestic studies have indicated that the normal inward angulation and forward tilt angle are approximately 30°, and any angle exceeding 40° or less than 20° is considered abnormal (11–13). Additionally, differences in cranial base structure may also be a potential significant factor influencing the spatial relationship between the SP and surrounding neurovascular structures. According to the head shape classification method proposed by Retzius, the head index is calculated using the formula: Head index = (maximum transverse diameter of the skull/maximum longitudinal diameter of the skull) × 100%. Based on this index, the skull can be classified into three types: dolichocephalic (head index <75%), mesocephalic (head index 75–79.9%), and brachycephalic (head index >80%).

Brachycephalic skulls have a shorter anteroposterior diameter of the cranial base, making the SP more likely to contact blood vessels or nerves during head rotation or swallowing, increasing the risk of compression. Mesocephalic skulls have relatively normal spatial structures, while dolichocephalic skulls have an elongated anteroposterior diameter, increasing the distance between the SP and important structures, thereby reducing the likelihood of compression (14). Based on this, the study retrospectively analyzed the imaging data of patients clinically diagnosed with Eagle Syndrome, selected key parameters such as SP length (L), inward angulation (*α*), forward tilt angle (*β*), and incorporating head shape (k). A comprehensive evaluation index (R) was proposed, with the formula: R = (L/30 + α/30 + β/30) × k, the k value is assigned according to head shape classification: dolichocephalic = 1.1, mesocephalic = 1.0, and brachycephalic = 0.9. The numerical values 30 mm and 30°are derived from common physiological reference values for length and angle, respectively, facilitating standardization across different measurement units. The aim of this study is to establish a more scientific and precise imaging diagnostic system for Eagle Syndrome, thereby improving the accuracy of clinical diagnosis.

## 2 Materials and methods

### 2.1 Study population

**Figure 1:**
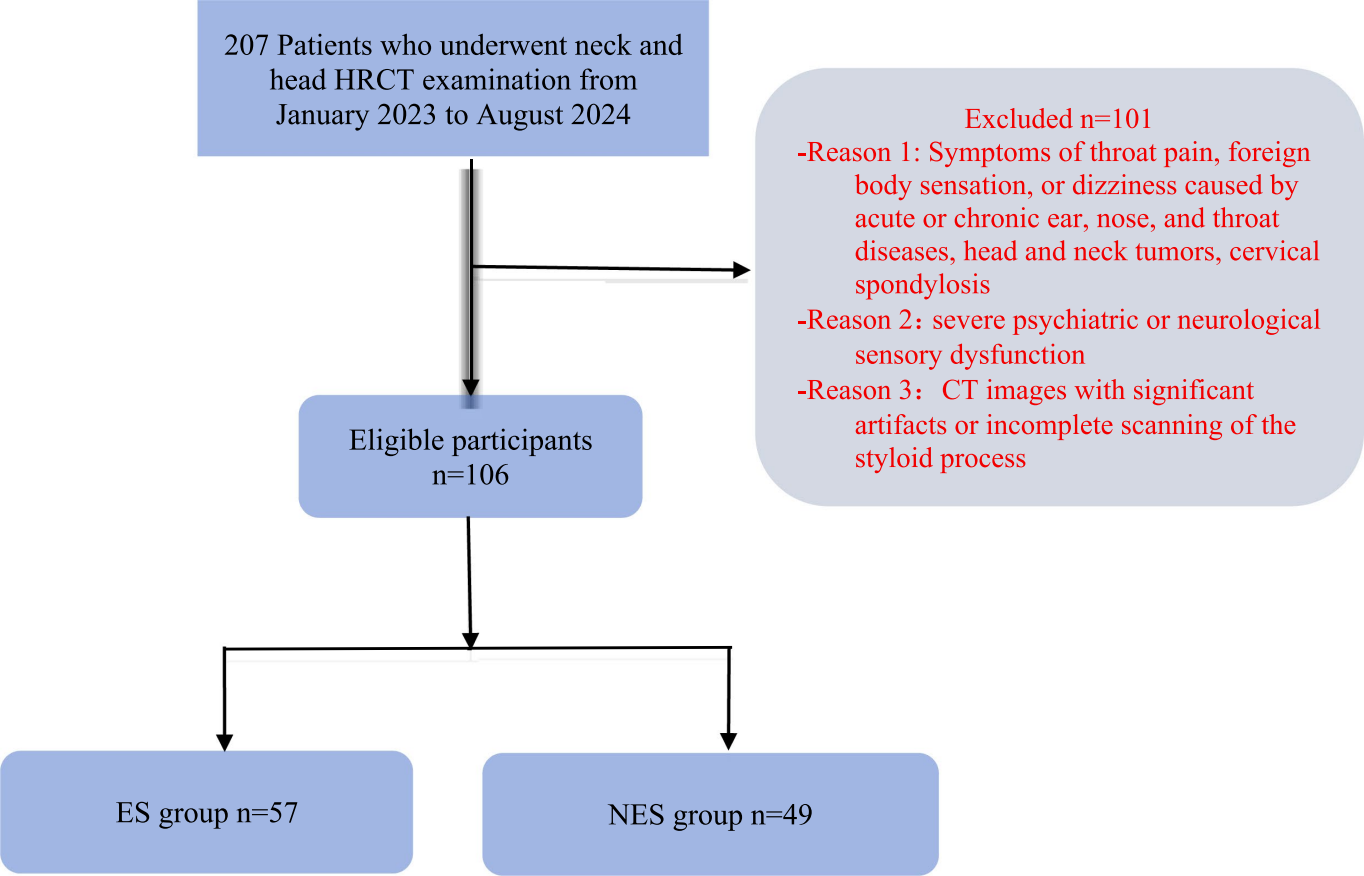
Flow chart of patient selection.

A total of 207 patients who underwent head and neck HRCT examinations at the Second Affiliated Hospital of Zhejiang Chinese Medical University between January 2023 and August 2024 were retrospectively collected. The patients sex, age at presentation, chief complaints, and HRCT imaging data were analyzed and recorded. Exclusion criteria: (1) symptoms such as pharyngeal pain, foreign body sensation in the pharynx, or vertigo after head rotation caused by acute or chronic otolaryngological diseases, head and neck tumors, or cervical spondylosis. (2) patients with severe psychiatric, neurological, or sensory dysfunction. (3) HRCT images with significant artifacts or incomplete coverage of the SP. A total of 101 patients were excluded, and 106 patients were finally included as study subjects. The ES group included patients who were eligible for the following Inclusion Criteria: (1) presence of at least one of the following chief symptoms—pharyngeal pain, foreign body sensation in the throat, vertigo on head rotation, anterolateral cervical pain, or radiating otalgia. (2) HRCT measurement of SP length >30 mm. (3) Age between 18 and 80 years. At last, a total of 57 patients were enrolled in ES group, involving 114 elongated SP, 35 males and 22 females, with a mean age of 56.89 ± 15.99 years. In contrast, the NES group comprised patients without the above mentioned symptoms, HRCT measurement of SP length <30 mm, and aged between 18 and 80 years. This NES group included 49 patients, involving 98 normal SP, including 22 males and 27 females, with a mean age of 55.08 ± 16.30 years. As follow in Figure 1. This study was approved by the hospital’s ethics committee (approval number: 2025-LW-083-01).

### 2.2 HRCT protocol and image processing

A 64-slice GE Light speed VCT scanner was used, with the scanning range from the inferior border of the mandible to the external auditory meatus, and scanning was performed from the feet to the head. Patients were instructed not to swallow during the scan. Scan parameters: detector arrangement 64 × 0.625, collimator width 0.625 mm, slice thickness 0.625 mm, pitch 1.0 mm, field of view 25 mm, tube voltage 120 kV, tube current 100–298 mA, window width/window level 1500/500 HU. The 0.625 mm thin slice bone window images were transmitted to a GE ADW 4.6 post-processing workstation for three-dimensional reconstruction. MIP was used to reconstruct the SP in its optimal shape. The images were measured for SP length and angles by two radiologists (J. D., a graduate student, and Q. R., an attending physician with 11 years of experience).

**Figure 2:**
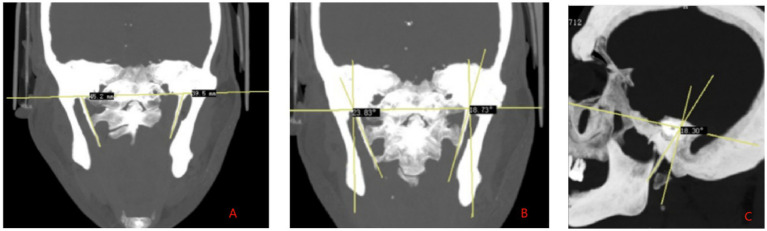
High-resolution CT three-dimensional reconstruction maximum density projection. **(A)** SP length measurement. **(B)** SP measurement of medial declination angle. **(C)** SP measurement of anterior inclination angle.

SP length measurement: The distance from the center of the SP root to the tip of the SP was measured on the CT reconstructed images, which is defined as the SP length (Figure 2A). SP angles: (1) Inward angulation: The reconstructed MIP image in the coronal plane clearly display the bilateral SP roots and tips. The angle between the long axis of the SP and the perpendicular line to the cranial base plane was measured (Figure 2B). (2) Forward tilt: The MIP image in the sagittal plane clearly showed one side of the SP and the inferior margin of the orbit, and the angle between the long axis of the SP and the inferior orbital margin and the internal ear cochlea was measured (Figure 2C). If the SP is curved or segmented during measurement, the long axis is defined as the line connecting the midpoint of the SP root and the midpoint of the distal end. Head index measurement: The maximum transverse diameter (biparietal diameter) and maximum anteroposterior diameter (occipitofrontal diameter) of the skull are measured. The head index is calculated using the formula: (transverse diameter of the skul/anteroposterior diameter of the skull) × 100 (15, 16).

### 2.3 Statistical analysis

Data analysis was performed using SPSS 27.0 and MedCalc (version 9.6.2.0) statistical software. Quantitative data were expressed as mean ± standard deviation (*x̄* ± s), and comparisons between groups were conducted using the independent samples t-test. Categorical data were expressed as frequency (percentage), and group comparisons were performed using the chi-square (χ^2^) test. To assess the inter-observer consistency of imaging data measurement metrics, with the following definitions: ICC < 0.4 indicates poor consistency. 0.4 ≤ ICC < 0.75 indicates moderate consistency. 0.75 ≤ ICC < 0.9 indicates good consistency. ICC ≥ 0.9 indicates excellent consistency. All statistical tests were two-tailed, and *p* < 0.05 was considered statistically significant. Pearson correlation analysis was performed for the SP length, inward angulation, and the diagnostic efficacy of the R value was assessed through Receiver Operating Characteristic (ROC) curve analysis, calculating the area under the curve (AUC) and its 95% confidence interval (CI), and determining the optimal diagnostic cutoff value. Finally, binary logistic regression was used to verify the stability of the model.

## 3 Results

### 3.1 The clinical, imaging characteristics, and R-value between the two groups

Table 1 summarizes the clinical characteristics between the two groups, showing no statistically significant difference in gender and age (*p* > 0.05). indicating that the two groups are comparable. The inter-observer consistency analysis within the groups revealed an ICC of 0.87, indicating good consistency between the two observers. Table 2 presents the results of the univariate analysis, summarizing the SP length, angle, head shape classification, and R-value for both the ES and NES groups, showing statistically significant differences between the two groups. In the ES group, the SP length was 34.19 ± 5.14 mm on the left side and 34.13 ± 6.40 mm on the right side, the inward angulation was 24.29 ± 3.09°on the left side and 22.22 ± 3.18°on the right side, and the forward tilt angle was 28.39 ± 2.76°on the left side and 28.29 ± 2.72°on the right side. In the NES group, the SP length was 23.78 ± 3.47 mm on the left side and 23.14 ± 3.29 mm on the right side, the inward angulation was 21.38 ± 5.45°on the left side and 20.46 ± 4.51°on the right side, and the forward tilt angle was 25.73 ± 1.45°on the left side and 25.56 ± 6.24°on the right side. In the ES group, the head shape classification was as follows: 7.02% (4/57) dolichocephalic, 28.07% (16/57) mesocephalic, and 64.91% (37/57) brachycephalic. In the NES group, the head shape classification was: 4.08% (2/49) dolichocephalic, 18.37% (9/49) mesocephalic, and 77.55% (38/49) brachycephalic. The R value in the ES group (left side 3.05 ± 0.26, right side 2.98 ± 0.31) was significantly higher than that in the NES group (left side 2.53 ± 0.35, right side 2.44 ± 0.31), with a larger R value indicating a higher probability of having Eagle Syndrome.

**Table 1:** Clinical features of ES and NES.

Clinical features	ES (*n* = 57)	NES (*n* = 49)	t/Chi^2^	*p*
Age	47.31 ± 14.47	58.98 ± 9.00	0.58	0.565
Gender			2.88	0.089
Male	35 (61.4%)	22 (38.6%)		
Female	22 (44.9%)	27 (55.1%)		

**Table 2:** CT features comparison among ES and NES: univariate analysis.

CT findings	Group	*N*	Mean ± standard deviation	t	*p*
LSL	ES	57	34.19 ± 5.14	12.35	<0.001^*^
	NES	49	23.78 ± 3.47		
RSL	ES	57	34.13 ± 6.40	11.34	<0.001^*^
	NES	49	23.14 ± 3.29		
LMD	ES	57	24.29 ± 3.09	3.32	<0.001^*^
	NES	49	21.38 ± 5.45		
RMD	ES	57	22.22 ± 3.18	2.29	<0.001^*^
	NES	49	20.46 ± 4.51		
LAI	ES	57	28.39 ± 2.76	2.84	0.003
	NES	49	25.73 ± 1.45		
RAI	ES	57	28.29 ± 2.72	4.44	<0.001^*^
	NES	49	25.56 ± 6.24		
LR	ES	57	3.05 ± 0.26	8.69	<0.001^*^
	NES	49	2.53 ± 0.35		
RR	ES	57	2.98 ± 0.31	9.02	<0.001^*^
	NES	49	2.44 ± 0.31		
Head shape classification				2.01	0.404
		
Dolichocephalic	ES	57	4 (7.02%)		
	NES	49	2 (4.08%)		
Mesocephalic	ES	57	16 (28.07%)		
	NES	49	9 (18.37%)		
Brachycephalic	ES	57	37 (64.91%)		
	NES	49	38 (77.55%)		

^*^p ≤ 0.05 is significant difference between groups. LSL: Left SP length. RSL: Right SP length. LMD: Left medial declination angle. RMD: Right medial declination angle. LAI: Left anterior inclination angle. RAI: Right anterior inclination angle. LR: Left R-value. RR: Right R-value.

### 3.2 Correlation analysis between SP parameters

There is a certain correlation between SP length and inward angulation as well as forward tilt, but the degree of association varies: the left SP length is weakly positively correlated with the left inward angulation (r = 0.203, *p* = 0.037), but there is no significant correlation with the left forward tilt angle (r = 0.153, *p* = 0.117). the right SP length show no significant correlation with the right inward angulation (r = 0.152, *p* = 0.119), but is weakly positive correlated with the right forward tilt angle (r = 0.227, *p* = 0.019). Additionally, no significant correlation was found between inward angulation and forward tilt (left: r = 0.078, *p* = 0.424; right: r = 0.162, *p* = 0.098), as shown in Table 3.

**Table 3:** Correlation analysis between SP parameters.

Variable	LSL		RSL		LMD		RMD		LAI		RAI	
	t	*p*	t	*p*	t	*p*	t	*p*	t	*p*	t	*p*
LSL			0.74	<0.001^*^	0.20	0.04	0.14	0.17	0.15	0.12	0.22	0.02
RSL	0.74	<0.001^*^			0.15	0.12	0.08	0.44	0.21	0.04	0.23	0.02
LMD	0.20	0.04	0.15	0.12			0.47	<0.001^*^	0.08	0.42	0.06	0.56
RMD	0.14	0.17	0.08	0.44	0.47	<0.001^*^			0.12	0.24	0.16	0.10
LAI	0.15	0.12	0.21	0.04	0.08	0.42	0.12	0.24			0.58	<0.001^*^
RAI	0.22	0.02	0.23	0.02	0.06	0.56	0.16	0.10	0.58	<0.001^*^		

^*^p ≤ 0.05 is significant difference. LSL: Left SP length. RSL: Right SP length. LMD: Left medial declination angle. RMD: Right medial declination angle. LAI: Left anterior inclination angle. RAI: Right anterior inclination angle.

### 3.3 Logistic regression analysis and ROC curves

**Figure 3:**
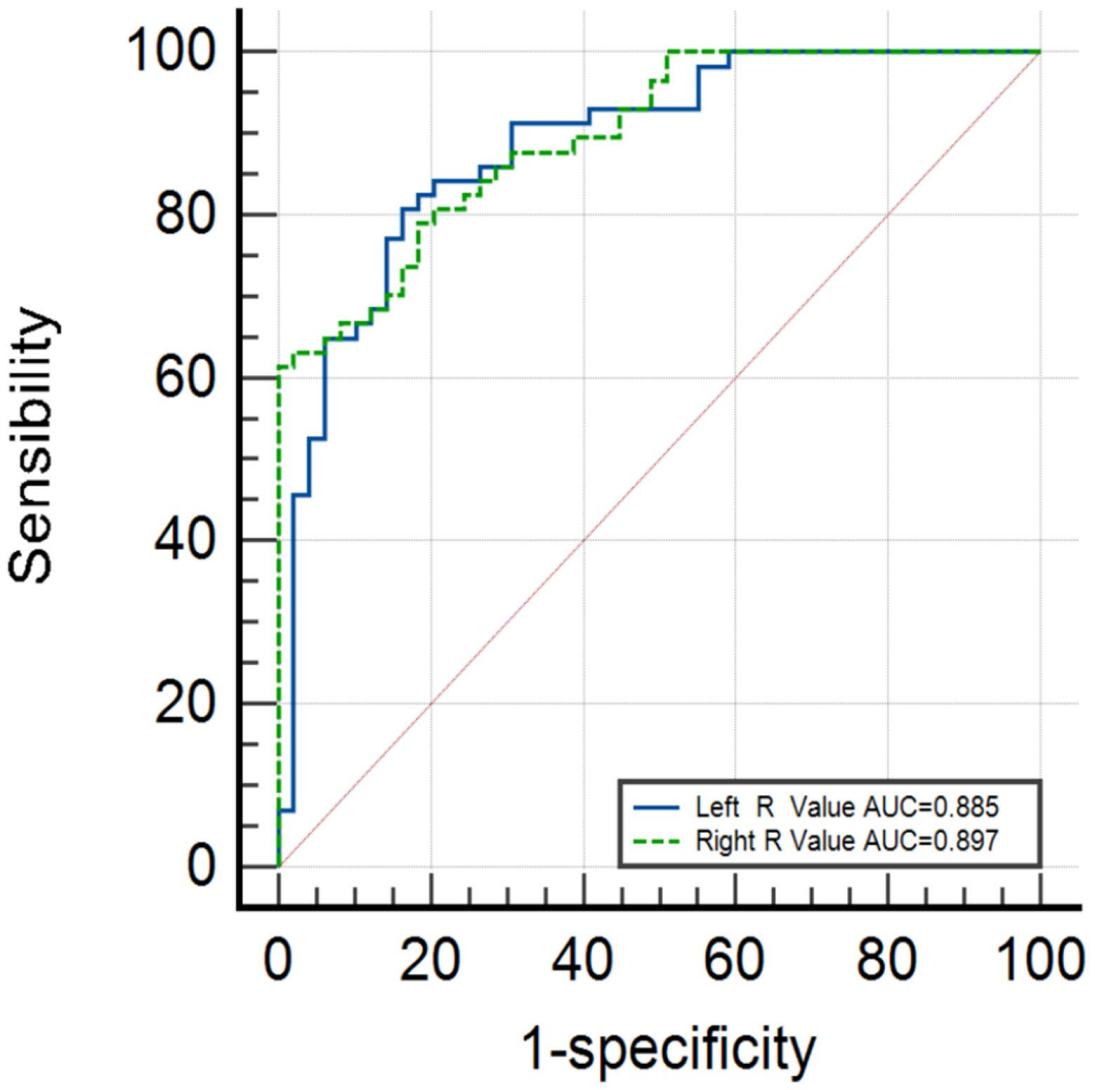
Initial R-value analysis.

**Figure 4:**
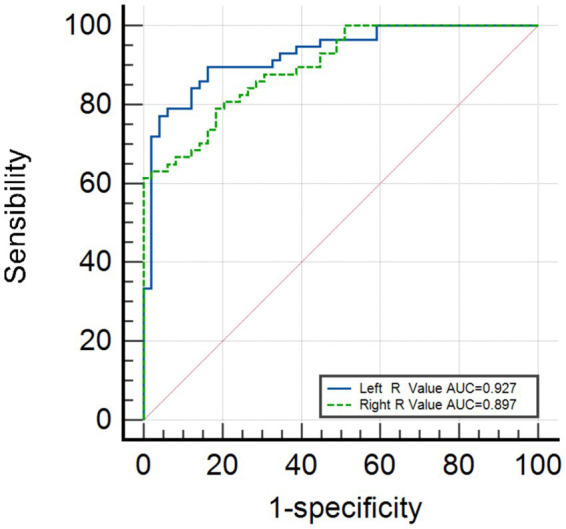
The logistics regression validation analysis.

Figure 3 shows that the area under the curve (AUC) for the left R-value is 0.885 (95% CI, 0.822–0.949), and the AUC for the right R-value is 0.897 (95% CI: 0.841–0.952). The optimal cutoff value for the left side is 2.86, with a sensitivity of 80.7% and a specificity of 83.7%. The optimal cutoff value for the right side is 2.89, with a sensitivity of 78.9% and a specificity of 81.6%. Logistic regression validation shows that the AUC for the left side is 0.927 (95% CI, 0.879–0.975), with a sensitivity of 89.5% and specificity of 83.7%, while the AUC for the right side is 0.897 (95% CI, 0.841–0.952), with a sensitivity of 78.9% and a specificity of 81.6%, as shown in Figure 4.

## 4 Discussion

The SP is a slender, cone-shaped bony protrusion located at the anteroinferior part of the temporal bone, originating from the tympanic part of the temporal bone and extending downward and forward. Due to its proximity to several important neurovascular structures, such as the internal carotid artery, external carotid artery, and glossopharyngeal nerve, its unique anatomical position makes it prone to mechanically compressing surrounding tissues when morphological abnormalities occur, thus triggering a series of clinical symptoms and leading to Eagle syndrome. The clinical manifestations of this syndrome are quite diverse and can involve neuropathic pain in the head and face, foreign body sensation in the throat, difficulty swallowing, and even symptoms of cerebral hypoperfusion. However, existing studies have shown that there is no simple linear relationship between the SP length and the severity of clinical symptoms (17, 18). Some individuals with significantly elongated SP may be asymptomatic, while others with only mild elongation may experience significant discomfort. This phenomenon suggests that in addition to length, the angulation of the SP may also play an important role in the pathogenesis mechanism (19, 20). Current research evidence indicates that an excessively inward angulation may compress the carotid artery, leading to symptoms such as tinnitus, headache, and even transient cerebral ischemia (21), while an increased forward tilt angle may stimulate peripheral nerves, such as the glossopharyngeal nerve, causing pain in the throat or neck (22). However, the correlation between the forward tilt angle and symptoms remains inconsistent in current studies, with some studies failing to confirm a direct association (23–26).

In this study, the majority of patients with Eagle Syndrome presented with symptoms such as throat pain and foreign body sensation in the throat. Due to the difficulty in differentiating throat pain and foreign body sensation caused by Eagle Syndrome from those caused by other diseases that can lead to neck or facial pain and abnormal sensations, we used HRCT scanning combined with three-dimensional reconstruction technology to systematically analyze the relevant anatomical parameters of 57 patients with ES and 49 NES. The results showed that three-dimensional reconstruction the SP in three-dimensional space, and provide a three-dimensional view of its spatial orientation and its anatomical relationship with surrounding tissues, offering important evidence for the diagnosis of Eagle Syndrome. There was no statistically significant difference in gender and age between the ES group and NES group, which is inconsistent with the conclusion of AlZarea (27) that the disease is more common in males. This discrepancy may be attributed to differences in genetic backgrounds and regional development characteristics. The aforementioned study primarily relied on data from Western populations, whereas our study sample was sourced from mainland China. This comparison suggests that SP characteristics may exhibit population heterogeneity, with differences in the developmental patterns of the SP across different regional populations. A recent large-scale cross-sectional study conducted on the population of Damascus, Al-Khanati et al. (28), revealed that the overall prevalence of SP elongation based on panoramic radiograph measurements was 4.5%. This figure closely aligns with Eagle’s initial estimate of 4%, but is much lower than the higher rates reported in studies from other regions. This comparison strongly suggests that ethnic, genetic, or regional factors may play an important role in the development and classification of the SP. Furthermore, the study reaffirmed the lack of a mandatory correlation between clinical symptoms and anatomical abnormalities: among the 179 cases of elongation detected, 88 were clinically followed up, and while most were symptomatic, about 8% of the elongated patients remained completely asymptomatic. This further supports the notion that diagnosis should not rely solely on imaging findings.

There are significant differences between the ES group and NES group in several key anatomical parameters, with bilateral SP length, inward angulation, and forward tilt angle being notably larger in the ES group compared to the NES group. Furthermore, these anatomical changes are significantly correlated with the occurrence of clinical symptoms, and the results are generally consistent with previous related literature (29). Further bivariate correlation analysis showed that SP length was positively correlated with the left inward angulation, left forward tilt angle, and right forward tilt angle, but there was no significant difference with the right inward angulation. This correlation between bilateral anatomical parameters is also in agreement with the results of multiple previous epidemiological studies (30, 31).

Traditionally, a length of 30 mm has been considered the threshold between a normal and elongated SP. However, increasing evidence suggests that this single standard has significant limitations. A morphological study based on HRCT by Muñoz-Leija et al. (24) provides strong support for this view. In their observation of asymptomatic individuals, they found that the average SP length in their sample exceeded the traditional 30 mm threshold, with up to 49.5% of individuals having SP length greater than 30 mm, yet none exhibited related clinical symptoms. The study further pointed out that it is the morphological classification and angulation of the SP, rather than its length alone, plays a more critical role in causing clinical symptoms. The findings suggest that angle might be an important, yet overlooked variable. This viewpoint is further supported by recent clinical research. A retrospective study by Bargiel et al. explicitly noted the essential difference between anatomical elongation and the onset of clinical symptoms (32). In their study, 76% of the SP on the untreated side exceeded 30 mm in length, with 48% exceeding 40 mm, but patients did not experience discomfort. This further confirms that “elongation” based solely on imaging is insufficient for diagnosing Eagle syndrome. The study also inferred from surgical outcomes that the most characteristic clinical symptoms of Eagle Syndrome are the sensation of a foreign body in the throat and throat pain associated with swallowing pain, while symptoms such as headache and neck pain showed significant improvement after surgery. In contrast, the relief rate of symptoms like tinnitus was lower. These findings strongly suggest that the nature of clinical symptoms and their causal relationship with the SP have greater diagnostic value than simple elongation measurements, highlighting the need for comprehensive morphological analysis.

From the perspective of craniofacial development, the baseline geometry of the cranial bones may also be a potential factor. Almuhawas et al. (33) demonstrated in their research that the human skull width reaches a plateau around the age of 20, after which it undergoes little significant change. Previous studies have observed that the length of the SP increases with age (34). Therefore, in addition to the morphological and angular parameters of the SP itself, the spatial relationship between it and the relatively constant cranial bone structure is likely a potential key factor in the onset of symptoms.

Based on the aforementioned theoretical assumptions, our study introduced the cranial index to quantify the morphology of the cranial base and preliminarily explored its association with Eagle Syndrome. Although the statistical significance of this association did not reach a significant level in the current sample, and the head index was not established as an independent risk factor in this analysis, this does not imply that the spatial structure of the cranial base can be disregarded. The introduction of the head index formula holds core value in providing us with a framework for quantitatively assessing the morphology of the cranial base. Even if its direct correlation is not significant, it still suggests that an individual’s cranial base anatomical structure is a fundamental variable in the spatial relationship between the SP and surrounding tissues. Based on both domestic and international literature, the normal inward angulation and forward tilt angle of the SP are both approximately 30°. If the angles exceed 40°or are less than 20°, they are typically considered abnormal. Therefore, in our study, 30°was established as the critical threshold for angular deviation. Our highlight lies in the integration of three key anatomical parameters: SP length, angle, and head shape. Based on these anatomical features, we have combined these dimensions through specific mathematical modeling to create a novel composite index for the first time, the R value. Diagnostic efficacy analysis shows that the model has excellent discriminative ability. Further logistic regression analysis confirmed the robustness of this model, providing an objective and quantitative assessment tool for the clinical diagnosis of Eagle Syndrome.

The limitations of our study are mainly as follows: First, the sample size is relatively small and the study is a single-center investigation. This may affect the generalizability of the study’s results. Future studies should aim to increase the sample size and conduct multi-center validation. Second, although an R value diagnostic model has been established, it has not yet been externally validated in an independent cohort, and its clinical applicability needs further confirmation. Future studies should focus on prospective research using large-scale multi-center data models. Third, most individuals in NES group were selected based solely on normal stem length, which may amplify the intergroup differences and affect the accuracy of the results. Fourth the measurement of the cranial index did not account for individual differences in cranial development, which may introduce measurement bias. Fifth, our R value model is relatively complex, and further research is needed to determine whether a simpler and more practical model can be developed to better address clinical issues. Lastly, beam hardening artifacts caused by the basal skull bone, especially the petrous part of the temporal bone in CT scans, may result in unclear display of the SP margins, posing challenges to the accuracy of SP length and spatial angle measurements.

## 5 Conclusion

The etiology of Eagle Syndrome is complex, and its manifestations are diverse. Diagnosis requires a comprehensive consideration of the relationship between structural abnormalities and clinical symptoms. Our study introduces the innovative quantitative indicator, R value, to integrate key parameters such as the length, angle, and head shape of SP. It provides an initial exploration into the diagnosis of Eagle Syndrome, offering certain clinical reference value for diagnosing Eagle Syndrome objectively and precisely.

## Data Availability

The raw data supporting the conclusions of this article will be made available by the authors, without undue reservation.
